# Long-term exposure and health risk assessment from air pollution: impact of regional scale mobility

**DOI:** 10.1186/s12942-023-00333-8

**Published:** 2023-05-19

**Authors:** Lorenza Gilardi, Mattia Marconcini, Annekatrin Metz-Marconcini, Thomas Esch, Thilo Erbertseder

**Affiliations:** 1 German Remote Sensing Data Center, Department Atmosphere (DFD-ATM), German Aerospace Center (DLR), Münchener Str. 20, 82234 Weßling, Germany; 2 German Remote Sensing Data Center, Department Land Surface Dynamics (DFD-LAX), German Aerospace Center (DLR), Münchener Str. 20, 82234 Weßling, Germany

**Keywords:** Exposure assessment, Air pollutants, Satellite-based data, Dynamic population, Diurnal variability, Settlement mask

## Abstract

**Background:**

The negative effect of air pollution on human health is widely reported in recent literature. It typically involves urbanized areas where the population is concentrated and where most primary air pollutants are produced. A comprehensive health risk assessment is therefore of strategic importance for health authorities.

**Methods:**

In this study we propose a methodology to perform an indirect and retrospective health risk assessment of all-cause mortality associated with long-term exposure to particulate matter less than 2.5 microns (PM_2.5_), nitrogen dioxide (NO_2_) and ozone (O_3_) in a typical Monday to Friday working week. A combination of satellite-based settlement data, model-based air pollution data, land use, demographics and regional scale mobility, allowed to examine the effect of population mobility and pollutants daily variations on the health risk. A Health Risk Increase (HRI) metric was derived on the basis of three components: hazard, exposure and vulnerability, utilizing the relative risk values from the World Health Organization. An additional metric, the Health Burden (HB) was formulated, which accounts for the total number of people exposed to a certain risk level.

**Results:**

The effect of regional mobility patterns on the HRI metric was assessed, resulting in an increased HRI associated with all three stressors when considering a dynamic population compared to a static one. The effect of diurnal variation of pollutants was only observed for NO_2_ and O_3_. For both, the HRI metric resulted in significantly higher values during night. Concerning the HB parameter, we identified the commuting flows of the population as the main driver in the resulting metric.

**Conclusions:**

This indirect exposure assessment methodology provides tools to support policy makers and health authorities in planning intervention and mitigation measures. The study was carried out in Lombardy, Italy, one of the most polluted regions in Europe, but the incorporation of satellite data makes our approach valuable for studying global health.

**Supplementary Information:**

The online version contains supplementary material available at 10.1186/s12942-023-00333-8.

## Background

The causal relationship between air pollution and the exacerbation of health outcomes, ranging from acute respiratory and cardiovascular diseases to chronic illnesses, has been comprehensively documented in medical literature [1, 2]. According to the European Environmental Agency, roughly 360,000 premature deaths could be attributed to the exposure to the main air pollutants in Europe in 2019 [3]. In 2020, 95%, 94% and 89% of the European urban population was exposed to concentrations of particulate matter less than 2.5 microns (PM_2.5_), nitrogen dioxide (NO_2_) and ozone (O_3_), respectively, exceeding the recommendations from the World Health Organization (WHO) [4]. Furthermore, the exacerbation of detrimental health endpoints from air pollution contributes to increase the susceptibility to infectious diseases [5]. To gain a better understanding of the related processes and interactions, enhanced tools are needed to enable planners and decision makers to effectively quantify the exposure of the population to air pollution.

A health risk assessment should include three components: the assessment of the hazard (i.e., the air pollution concentration), the vulnerability of the individuals (i.e., the dose–response functions) and the probability of exposure [6–8]. The latter cannot be assessed by air pollutant levels alone [9]. In fact, neglecting the mobility pattern can lead to systematic errors in air pollution exposure and health burden assessments [10, 11]. An exposure assessment can be accomplished at the individual level using surveys, wearables, microsensors, or mobile data [10, 12–14]. Besides Geographic Information System (GIS)-based modelling approaches [15], data from Global Positioning System (GPS) devices [16], such as mobile phones, have been successfully exploited for a dynamic assessment of exposure to air pollution [10]. Despite the valuable results of studies implementing these systems, they can only provide a temporally and spatially limited snapshot of the complex reality; extrapolations to long-term effects and other areas are prone to large uncertainties. Population mobility patterns can also be assessed with self-reported household travel surveys [17, 18]. Major disadvantages of this approach are the large non-response rate, the non-representativeness of samples and the high costs [18, 19].

For the above-mentioned reasons, many health risk assessments and epidemiological studies assume a static population, i.e. the air pollution concentration at the geolocation of the residential address is considered [20–22]. Another possible solution to assess the exposure is the definition of so-called microenvironments. This simplification of the real world allows for the clustering of exposure scenarios with similar features, to which the population is dynamically assigned on the basis of typical time patterns [9, 23, 24].

However, recent studies assessing the health risk from air pollution incorporating assumptions on population dynamics and making use of microenvironments are often confined to single cities, short time periods, single air pollutants and the use of in-situ measurements [17, 25, 26]. Hence, the representativity of these studies to draw general conclusions is limited [9]. The challenge is therefore to fill this data and methodological gaps with a scalable approach that can be easily applied to multiple areas of interest. In particular, to meet the requirement for global health studies a worldwide applicable method is necessary.

To fill these gaps, we present a scalable method based on a combination of data assets, to quantify the long-term population exposure to air pollutants and to perform a health risk assessment, considering the daily variation of multiple pollutants and the population dynamics. The resulting effects were examined by two metrics: the Health Risk Increase (HRI) and the Health Burden (HB).

## Methods

### Outline

HRI due to PM_2.5_, NO_2_ and O_3_ was quantified using a top-down approach, introducing a combination of satellite remote sensing imagery, mobility patterns, dose–response functions, infiltration ratios and chemical transport modelling data. Diurnal air quality variability was obtained from hourly data of the multi-year reanalysis of the Copernicus Atmospheric Monitoring Service (CAMS) [27, 28]. The Fraction of Settled Area (FSA) was calculated from the World Settlement Footprint 2019 (WSF 2019) dataset [29] and used as a proxy for the probability of population’s presence. Indoor/outdoor concentration’s ratios (IOR) were taken from the literature [30, 31] and assigned to areas marked as “settled-building” in the WSF 2019. Relative Risk (RR) values from the WHO were utilized [32]. These data layers were combined to quantify the HRI of all-cause mortality, associated with the long-term exposure to air pollutants during the day and the night hours. The expression “all-cause mortality” refers to natural causes of death. Accidental or violent causes of deaths are therefore excluded. In the study, we compared the exposure of population to increasing HRI ranges in a static and in a dynamic population scenario.

**Fig. 1:**
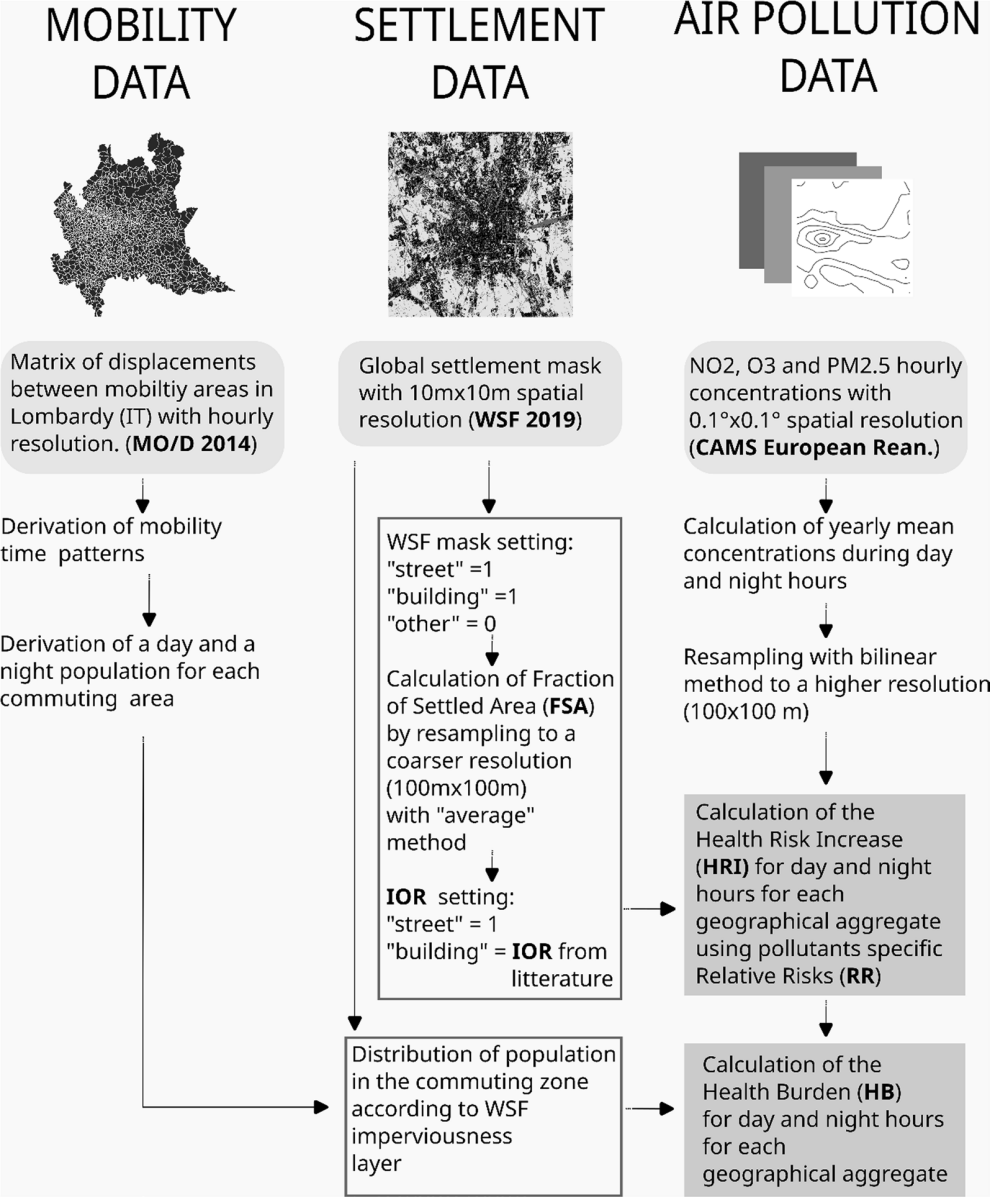
Conceptual representation of the data processing and derivation the Health Risk Increase and the Health Burden metrics

The Health Burden (HB) aims at providing a measure of the potential impact of the exposure to air pollution on the economic and sanitary systems. This metric considers the number of people and their diurnal mobility patterns, obtained from publicly available mobility data for the Lombardy region, the “Matrice Origine/Destinazione 2014” (MO/D 2014). To build it, the novel WSF 2019 was employed [29, 33], which proportionally redistributes population figures available at the finest possible administrative level using local imperviousness and land-use information gathered from OpenStreetMap [34]. The data processing and methodology adopted in our study are outlined in Fig. 1. Information on all data layers and a detailed explanation of the HRI and HB metrics can be found later in this chapter.

### Study area

**Fig. 2:**
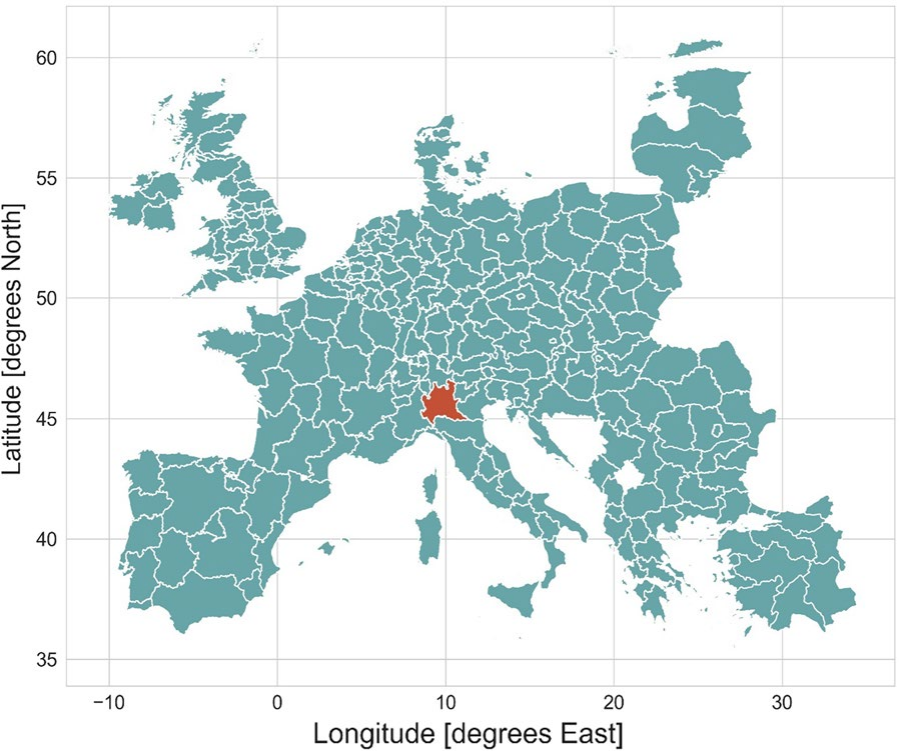
Geographical location of the study area Lombardy, Italy

The area of interest is Lombardy, in northern Italy, see Fig. 2. The location is particularly suitable for the intended study for a number of reasons. The region is one of the most densely populated areas in Europe: it hosts about one sixth of the total Italian population (around ten million people) and accounts for about one fifth of the share of the national gross domestic product [35]. The region is highly industrialized. The Po Valley is historically known to be susceptible from an air quality perspective. Due to its peculiar morphology, where the Alpine and Apennine mountain ranges limit the diffusion in the boundary layer [36, 37], high pollution levels are frequently reported. Despite the general emissions reduction following the strict lockdown measures implemented in Italy in 2020, mean yearly pollution concentration values, above the WHO air quality guidelines, have still been recorded for the area [4]. Furthermore, for the Lombardy region, datasets of population mobility are publicly available.

### Air pollution data

CAMS is a service part of the Copernicus Program that provides hourly data on the regional atmospheric composition. The product consists of an ensemble of seven regional chemical transport models that have been increased to nine models after the upgrade performed in 2019 [28, 38]. Every day, analyses and forecasts data of the main pollutants are released with an hourly temporal resolution and a horizontal spatial resolution of 0.1 x 0.1 degrees.

The present study used CAMS reanalysis hourly data for the surface level of concentrations PM_2.5_, NO_2_ and O_3_ from 2014 to 2018. These where the most recent reanalyses data available at the time of the study. They are derived from near-real time datasets of previous years by means of the assimilation of and validation with in-situ observations from the European Environmental Agency.

### Population density and mobility data

Mobility data for the Lombardy region were obtained from the “Matrice Origine/Destinazione 2014”, a dataset developed within the regional mobility and transport program of Regione Lombardia. This is a matrix that presents the number of hourly displacements on a typical weekday within and between 1450 defined mobility zones in Lombardy. The matrix considers 8 modes of travel (e.g. car-driver, car-passenger, on foot, by bicycle, etc.) and 5 different motivations (e.g. study, work, occasional, etc.). The information is the result of the complex interaction between transport modelling, on-line questionnaires, face-to-face interviews carried out at railway and road borders, analysis of available surveys and of the existing demand detected [39]. It was built based on a transport model integrating the results of a survey held from February to May 2014 with data from the “2011 Census” of the Italian National Institute of Statistics (ISTAT) and with contributions from local authorities and stakeholders from the mobility sector. The number of permanent residents for each mobility zone is also available in the matrix and derived from the “2011 ISTAT Census”.

**Fig. 3:**
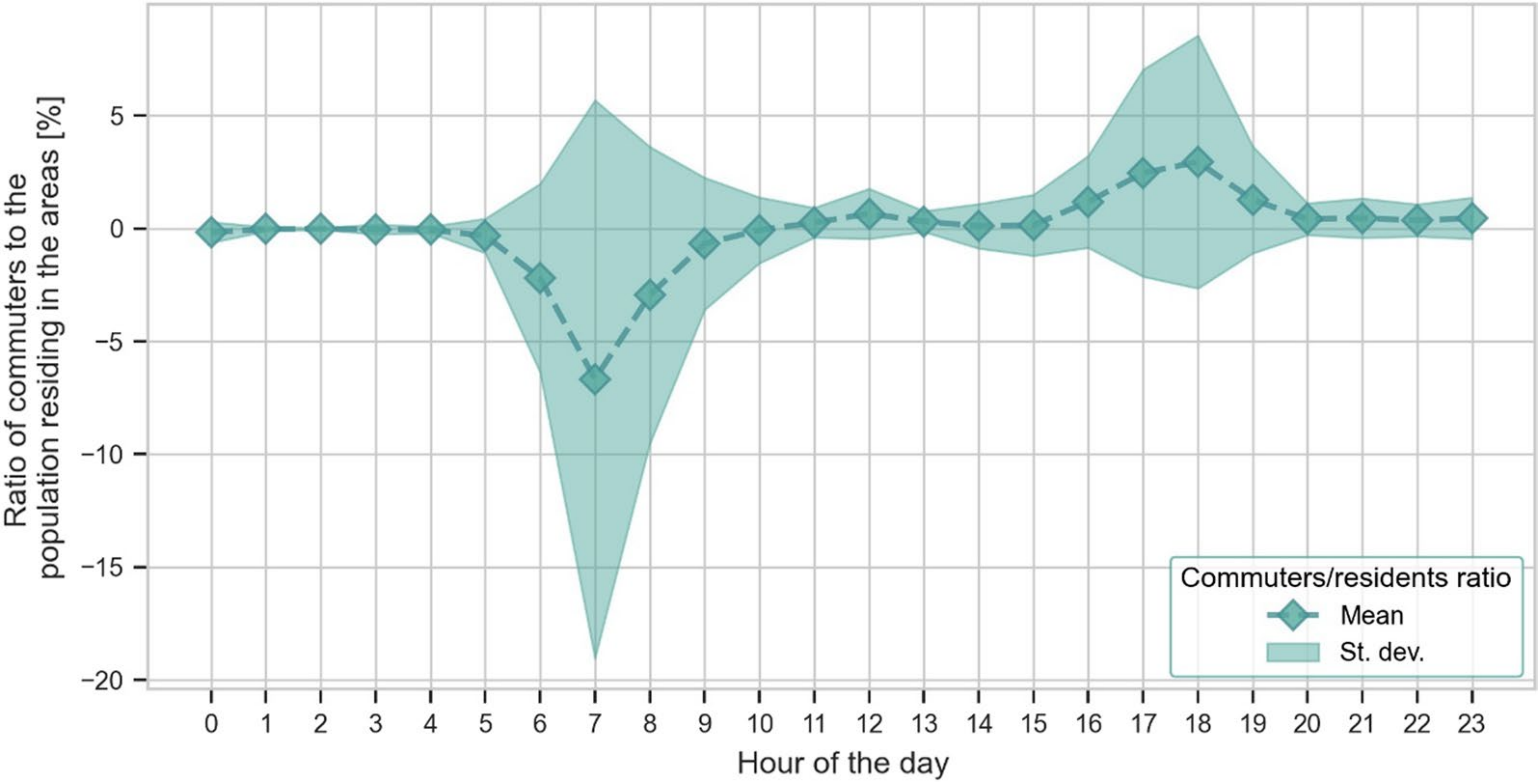
Ratio of net commuters and resident population averaged over Lombardy (Italy) for the different hours of the day. The dashed line represents the mean while the shaded area represents the standard deviation. Most of the displacement occurs between 6 and 9 a.m. and between 4 and 7 p.m

For this work, the data of the matrix were elaborated in order to obtain, for each hour of the day, the difference between the total number of displacements to/from each mobility zone, which represents the area-specific net number of commuters. The percentage in relation to the resident population was then obtained and the mean commuters’ oscillation across Lombardy and during the different times of the day was derived. The result is illustrated in Fig. 3.

**Fig. 4:**
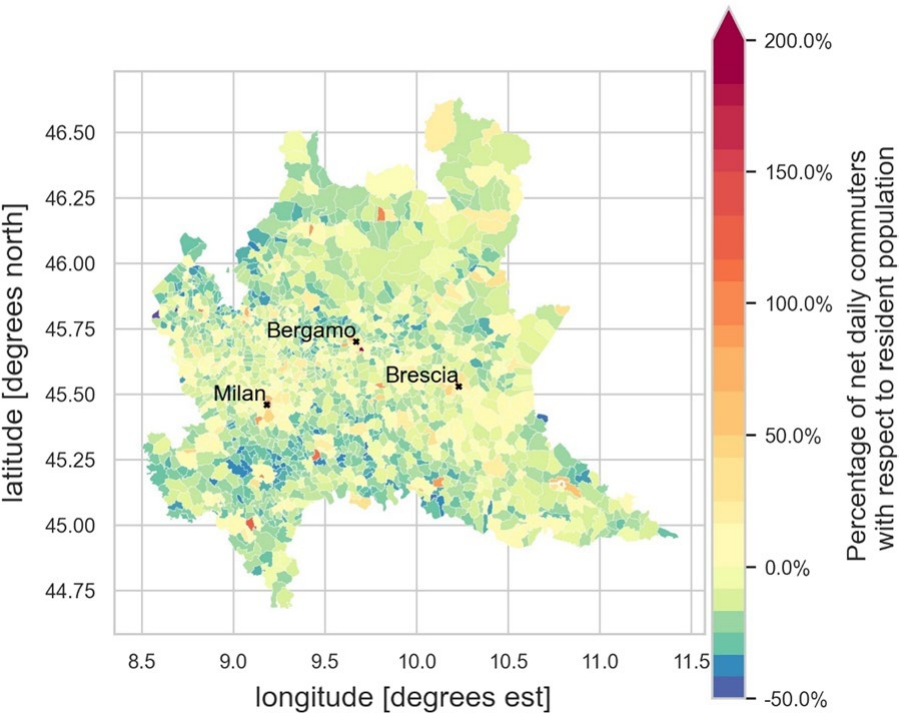
Percentage of net daily commuters traveling to/in different mobility zones with respect to the area’s resident population in the Lombardy region (Italy). Orange to red colours represent an increased daily population with respect to the resident one while green to blue colours represent a decrease

Most travel takes place between 6 and 9 a.m. and between 4 and 7 p.m. In these time ranges, the net commuters increase the residents of 2%. Between 9 a.m. and 4 p.m., the population of the areas is stable. Figure 4 shows a spatial visualization of the percentage of net daily commuters traveling to/from different mobility areas in relation to the resident population.

Based on this observational data set, two scenarios were elaborated: a “DAY” scenario, where the population of the areas is given by the residents plus the net commuters, calculated considering the population at 3 p.m., before the start of the back-commuting and a “NIGHT” scenario, with residents only.

### Population density and world settlement footprint

In order to assess the population exposure within the mobility zones, two data layers were derived. The first, the Fraction of Settled Area, was obtained from the World Settlement Footprint, a global settlement extent mask derived at 10 meters spatial resolution by jointly exploiting multitemporal satellite imagery from Sentinel-1 to Sentinel-2 [29]. The layer classifies each pixel as “non-settled”, “settled-building” or “settled-road”. The FSA was derived assigning a unitary value to settled pixels and zero to non-settled ones and performing an “average” resampling to 100 m spatial resolution. The FSA is used as a proxy for the probability of population presence.

The second layer is a population distribution layer for the “DAY” and “NIGHT” scenarios. For this purpose, the novel WSF 2019 population layer was employed, which estimates, for each 10 meters resolution pixel marked as settlement in the WSF 2019, the corresponding number of inhabitants. Specifically, this was obtained by proportionally redistributing the population figures derived from the MO/D 2014 by means of the local imperviousness, i.e. a reliable proxy for the built-up density [40], as well as land-use information gathered from OpenStreetMap. In the two scenarios, the total population was distributed to areas classified with specific land use labels. In the NIGHT scenario, from 8 p.m. to 6 a.m., the resident population only was distributed across areas labelled as “Residential”. During the DAY scenario, from 6 a.m. to 8 p.m., the population composed by residents plus/minus the commuters was distributed over areas classified as “Residential”, “Industrial” and “Commercial”.

### Calculation of the health risk increase

The hourly pollution data layers were oversampled in order to match a grid with resolution of 100 × 100 meters and interpolated using a bilinear method. Yearly aggregates of mean PM_2.5_, O_3_ and NO_2_ concentrations were calculated for the area of interest from the CAMS reanalysis data, separately for DAY and NIGHT hours. For PM_2.5_ and NO_2_ the yearly mean concentration was derived. For O_3_ a different metric was considered. Specifically, since O_3_ concentration presents relevant diurnal and seasonal fluctuations, the peak season aggregate was calculated for each year. This is obtained by performing the average of daily maximum in an 8-h rolling mean in the 6 consecutive months of the year, with the highest 6-month running-average O_3_ concentration [32, 41]. Finally, multiyear means between 2014 and 2018 were calculated.

Indoor/Outdoor Ratio coefficients, specific for each pollutant, obtained from the study of Monn et al. [30] and Cyrys et al. [31], were applied in correspondence to pixels classified as “settled-building” in the WSF 2019. A unitary coefficient was applied to pixels classified as “settled-street”. The IOR adopted for NO_2_, O_3_ and PM_2.5_ were, respectively: 0.8 (ref. [30]), 0.8 (ref. [30]) and 0.7 (ref. [31]). It is important to underline that these values do not consider indoor sources. The risk assessment therefore refers only to the contribution to health effects from outdoor sources. For each IOR coefficient, a range of values has been provided in literature. From these ranges, the highest value was considered in this study, in order to work according to a worst-case scenario.

The vulnerability to air pollutants on the increased all-cause mortality was determined using the dose–response functions provided by the WHO and expressed in terms of RRs. These are listed values derived by epidemiological studies, accompanied by their 95% Confidence Interval (CI). The WHO has recently released new RR values obtained through a meta-analysis conducted on the existing literature and published within the WHO air quality guidelines in 2021 [32]. They quantify the increase of the probability of a health outcome for a group exposed to an increased concentration of 10 µg/m^3^ of pollutants compared to the probability of a control group. The ratio of these two probabilities is the relative risk, see Eq. 1. If the RR is greater than one, the exposure to the factor considered is detrimental to the health. If the RR is smaller than one, it is beneficial. To be considered statistically significant, the CI should not range across positive and negative values [42]. For this study, RRs for all-cause mortality associated with the long-term exposure to PM_2.5_, NO_2_ and O_3_ were used. The considered values with their 95% confidence interval were 1.08 [1.06–1.09], 1.02 [1.01–1.04] and 1.01 [1.0–1.02], respectively. For PM_2.5_ and NO_2_ these values refer to the yearly mean concentration while, for O_3_, to the yearly seasonal peak metric, as described by the WHO [32]. In accordance with the approach adopted by the WHO, a linear dose–response relationship was assumed. However, as reported in the WHO Air Quality Guideline 2021 [32], a supralinear behavior can be expected at low concentrations, suggesting a steeper risk increase at lower exposure levels. Population exposure was quantified indirectly, using the Fraction Settled Area as a proxy variable and obtained from the WSF 2019. This was used as a measure the probability of human presence. Finally, the Health Risk Increase was calculated by multiplying the three components for each grid-cell in the domain, as shown in Eq. 2. The HRI was thus obtained for both the DAY and NIGHT scenarios. Geographical aggregates of the HRI were derived for the commuting areas.

1 $$RR\text{=} \, \frac{\text{Probability } \, {\text{of}} \, \text{ disease } \, \text{with } \, {\text{exposure}} \, }{\text{Probability } \, \text{of } \, \text{disease } \, \text{without } \, {\text{exposure}}}$$

where: RR: Relative risk of mortality referred to a concentration of 10 µg/m^3^

The Probability of the disease with and without the exposure to the target factor is provided as relative frequency of the health outcome in the exposed group and in the control group.

Relative risks do not deliver information on the absolute risk of a certain health outcome but provide information on the increased or decreased likelihood of a certain health event, given an exposure to an external factor. This increased/decreased likelihood is expressed by the distance of the resulting relative risk from the unit value, corresponding to RR-1.

2 $$HRI=C*\frac{RR-1 }{10} *FSA*IOR$$

where: RR: Relative risk of mortality referred to a concentration of 10 µg/m^3^. C: Pollutant concentration in µg/m^3^. FSA: Fraction settled areas from WSF. IOR: Indoor/outdoor ratio (this is posed equal to 1 for pixels labelled “street”)

In line with the WHO assumption of a linear dose–response relationship, the risk increase is obtained by multiplying the pollutant concentration by the slope coefficient (RR -1)/10. The normalization by 10 is adopted because RR values by WHO are given for an incremental pollutant concentration of 10 µg/m^3^. The pollutant concentration is corrected by the IOR factor for pixels labelled as “building”. Finally, the value is weighted by the FSA, a number between 0 and 1, which is used as a proxy for the probability of human presence.

### Calculation of the Health Burden

The HB metric has been formulated in order to provide a measure of the potential economic and public health impact. This metric considers the number of people and their mobility patterns. As illustrated in Fig. 1, it was derived by multiplying pixelwise the HRI by the total number of inhabitants (P), obtained from the WSF 2019 population layer for the day and night scenarios, as illustrated in Eq. 3. The population group considered for the calculations included, in the DAY scenario, the residents plus/minus the commuters and, in the NIGHT scenario, the residents only. Geographical aggregates of the HB were derived for the commuting areas.

3 $$HB=HRI*P$$

## Results

**Fig. 5:**
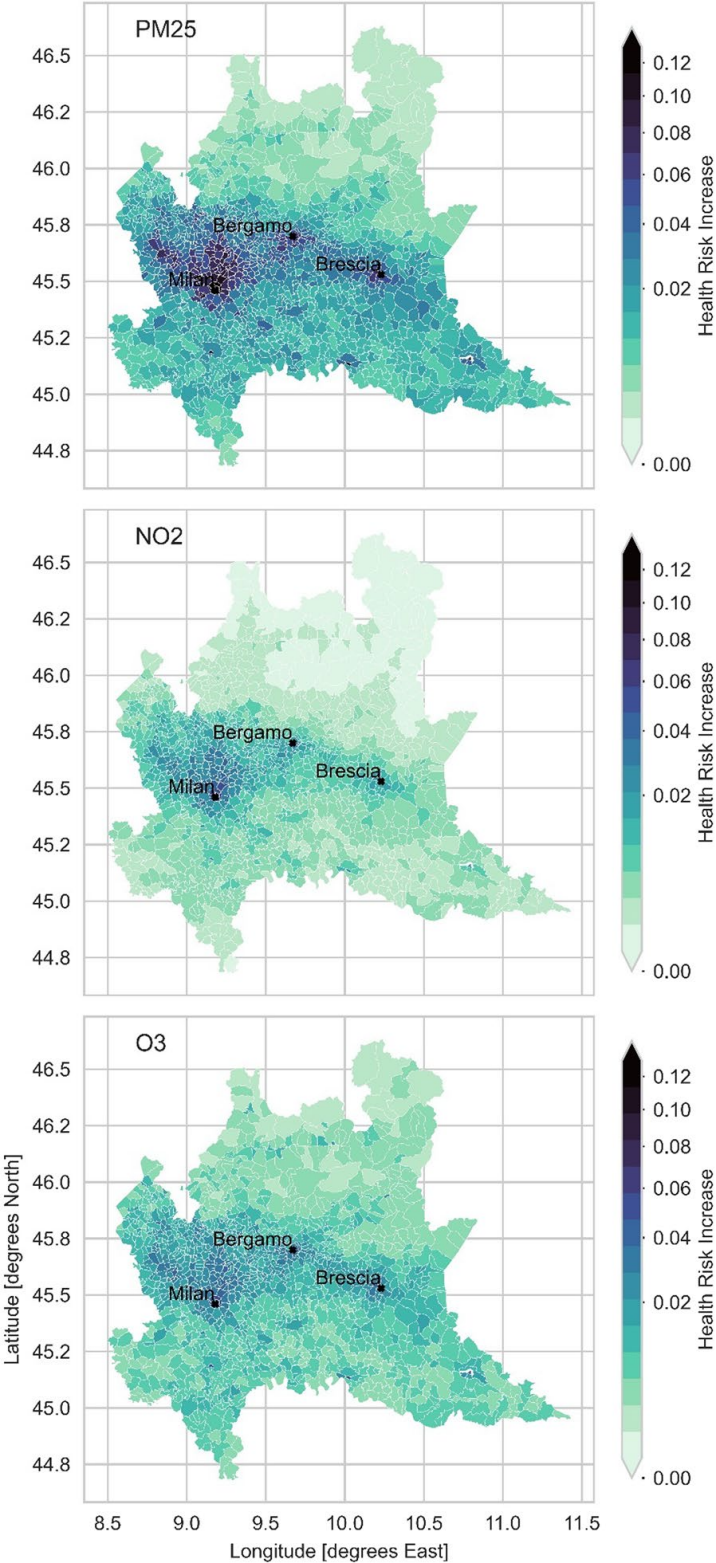
Multiannual mean Health Risk Increase (HRI) of all-cause mortality for daily hours scenario, associated with the exposure to PM_2.5_, NO_2_ and O_3_

The HRI and the HB associated with the all-cause mortality were quantified for the years from 2014 to 2018 in relation to O_3_, PM_2.5_ and NO_2_ concentrations in the day and in the night hours. Mean and maximum were determined for small geographical aggregates (i.e. the commuting zones) utilized in the mobility matrix dataset. The multi-annual mean HRIs for PM_2.5_, NO_2_ and O_3_ are reported for the DAY scenario, with the highest HRIs associated with PM_2.5_. The results are reported in Fig. 5. The highest HRI values, up to 12.5%, were observed in metropolitan areas around Milan, Mantua and Brescia for both DAY and NIGHT scenarios. A similar pattern was observed for HRI associated with O_3_ during the day, with more modest values exceeding 5% in commuting areas of several cities. The highest daily HRI daily values for NO_2_, up to 5.5%, were highly localized over urban areas.

Table 1 displays a summary of the statistical aggregates of the multi-year HRI, calculated for the whole of Lombardy, for the DAY and NIGHT scenarios. A T-Student test was performed to compare the mean multiannual HRI for each pollutant in the DAY and NIGHT scenarios. The results showed that the DAY HRI for NO_2_ and O_3_ was significantly lower compared to the NIGHT scenario. No significant result could be derived for PM_2.5_.

**Table 1:** Mean day and night statistical aggregates for Lombardy obtained from the multiyear geographical mean, minimum and maximum values of HRI of the commuting areas

	NO_2_	O_3_	PM_2.5_
**Day**			
Min	0.001% (0.0006–0.003)	0.01% (0.0–0.003)	0.004% (0.003–0.004)
Mean	0.76% (0.38–1.52)	1.23% (0.0–2.46)	2.32% (1.74–2.61)
Max	3.29% (1.64–6.58)	6.4% (0.0–12.76)	11.31% (8.48–12.72)
**Night**			
Min	0.001% (0.0006–0.003)	0.002% (0.0–0.003)	0.004% (0.003–0.004)
Mean	0.87% (0.44–1.75)	1.32% (0.0–2.65)	2.34% (1.75–2.63)
Max	3.88% (1.94–7.76)	6.91% (0.0–13.82)	11.46% (8.59–12.89)
**p-value**			
	0.0001*	0.0033*	0.56*
	H_0_ rejected	H_0_ rejected	H_0_ can’t be rejected

In brackets the values obtained using the 95% confidence interval values of the relative risks as denoted. In the last two rows, the results from the T-test comparing the HRI obtained for the DAY and the NIGHT scenarios, are shown

*One-tailed T-test with null hypothesis H_0_: µ ≥ µ_0_. The mean daily HRI is equal or greater than the mean night HRI

Additional file 1: Table S1 shows the top ten commuting zones with the highest multi-annual mean of HRI for PM_2.5_, NO_2_ and O_3_ during the day and night. The geographical classification is based on the MO/D 2014 data.

**Fig. 6:**
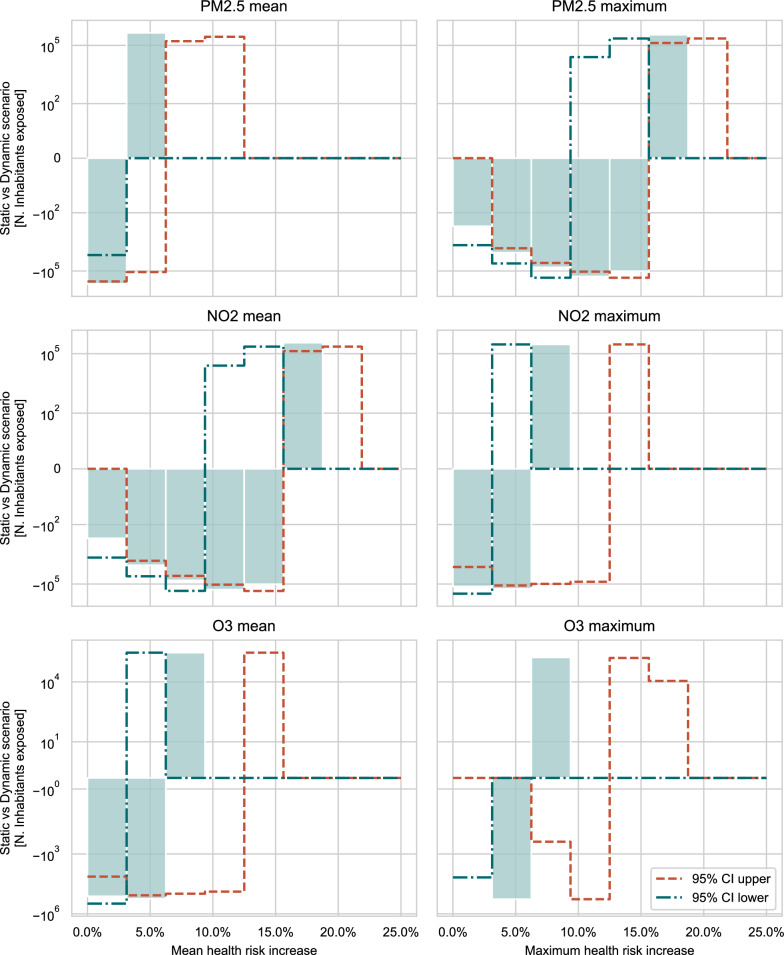
Number of people exposed to different ranges of mean (left column) and maximum (right column) HRI of all-cause mortality, associated with the long-term exposure to PM_2.5_, NO_2_ and O_3_. The solid histogram bars represent the HRI calculated using the RR values provided by the WHO, the green dashed-dotted line represents the HRI calculated using the RR’s 95% CI lower limit, while the red dashed line, the one using the upper the 95% CI upper limit

The difference in the number of people exposed to different HRI ranges is investigated by comparing the static (i.e., residents) and dynamic (i.e., residents plus/minus commuters) population for the DAY scenario using the MO/D 2014 mobility data. The analysis was performed for the 95% CI for each pollutant-specific relative risk, too. The results, shown in Fig. 6, indicate that a larger portion of the population is exposed to higher HRI ranges in the dynamic population scenario, particularly for maximum HRI aggregates, with a difference of hundreds of thousands of inhabitants. For PM_2.5_, 418,649 people were found to be exposed to higher multiannual mean HRI while for O_3_ and NO_2_ the numbers are 415,010 and 350,694 respectively. For maximum HRI aggregates these values correspond to 386,464, 29,107 and 257,223.

**Fig. 7:**
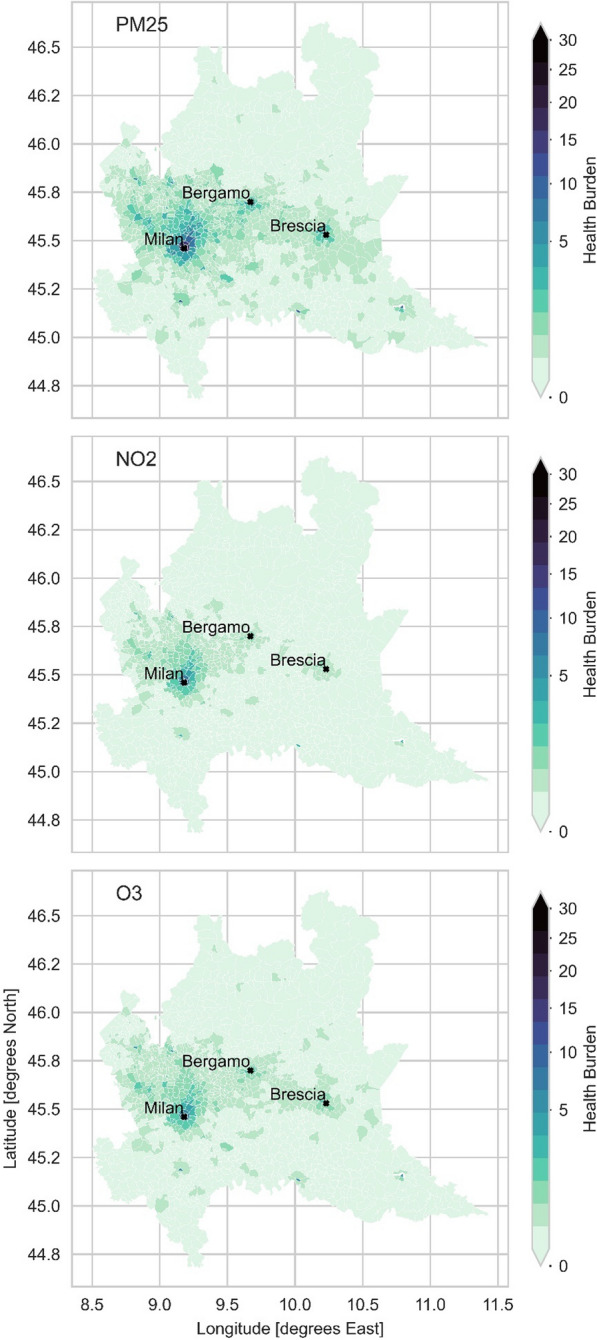
Multiannual mean Health Burden of all-cause mortality associated with the exposure to PM_2.5_, NO_2_ and O_3_ in the daily hours

**Fig. 8:**
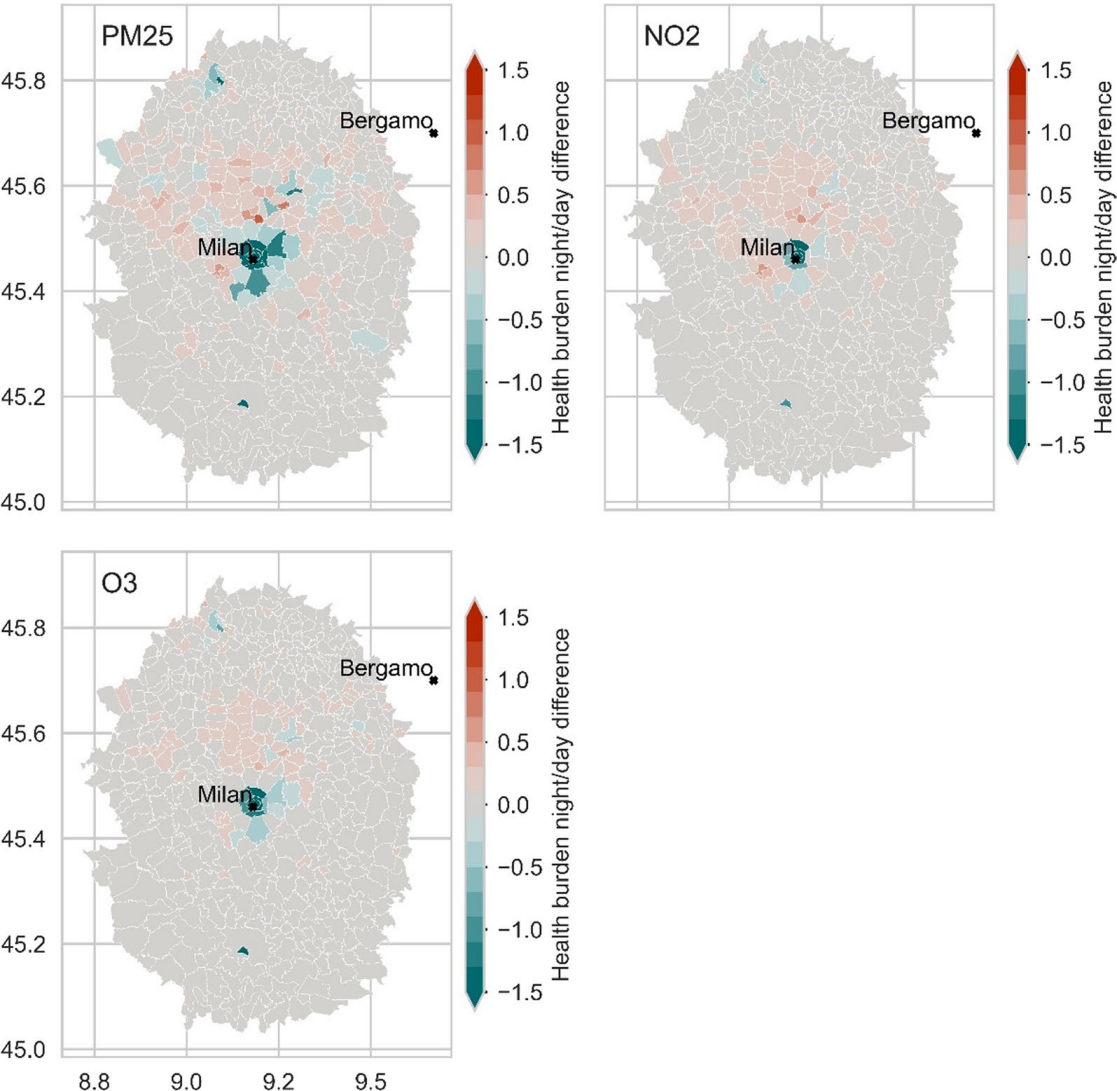
Difference of multiannual Health Burden between the night and day scenarios due to exposure to PM_2.5_, NO_2_ and O_3_: zoom over the Milan region with a radius of 0.4° from the city centre

The results for the Health Burden are displayed in Table 2 and Fig. 7. The population size is the most important factor influencing the HB score. In most of Lombardy, during the DAY scenario, the HB score is close or equal to zero. Higher values are only observed in urban areas, especially for NO_2_. The results for O_3_ and PM_2.5_ show low to moderate values over a wider area. A comparison of the HB scores between the DAY and NIGHT scenarios reveals the impact of back-commuting to the area of residence on the final result. Major urban centers and industrial clusters show a negative difference between the NIGHT and DAY scenarios, which is particularly visible in Milan and its surroundings. In Fig. 8 a view within a radius of 0.4 degrees around Milan is provided.

**Table 2:** Mean day and night statistical aggregates for Lombardy obtained from the multiyear geographical mean, minimum and maximum values of HB of the commuting areas in Lombardy

	NO_2_	O_3_	PM_2.5_
**Day**			
Min	0.0	0.0	0.0
Mean	20.0	26.7	53.8
Max	156.5	271.5	465.9
**Night**			
Min	0.0	0.0	0.0
Mean	20.7	26.6	49.8
Max	239.9	382.9	625.4
**p-value**			
	0. 38*	0.51*	0.76*
	H_0_ can’t be rejected	H_0_ can’t be rejected	H_0_ can’t be rejected

In the last two rows the results from the T-test when comparing the HRI obtained for the DAY and the NIGHT scenarios

*One-tailed T-test with null hypothesis H0: µ ≥ µ0. The mean daily HB is equal to or greater than the mean night HB

A T-student test was performed between the multi-annual mean HB values for PM_2.5_, NO_2_ and O_3_ during the day and night, using a one-tailed T-test. The null hypothesis assumed a mean of the day distribution greater than or equal to the mean of the night distribution. The p-value in all three cases was greater than 0.05 and, therefore, no significant conclusion could be drawn. The top-10 commuting areas with the highest Health Burden associated with PM_2.5_, NO_2_ and O_3_ for the day and night scenarios are presented in Additional file 2: Table S2.

## Discussion

### Key findings

Mobility data from the MO/D 2014 were elaborated in order to find the mean time pattern of the displacements between the commuting areas in Lombardy, Italy and to define a DAY and a NIGHT scenario. It was found that, on average, the commuting areas have a stable population between 8 p.m. and 6 a.m. and between 9 a.m. and 4 p.m. The majority of travels takes place in the complementary timeframes. For the three pollutants considered, the long-term daily HRI calculated assuming a dynamic population increases considerably compared to a static population scenario. This is consistent with the fact that, during the day, people that commute for studying and working purposes tend to travel to main urban centres, where commercial and industrial activities are concentrated. As a result, the daily population density in these areas is much higher than the nominal one, that counts only the residents. DAY HRI for NO_2_ and O_3_ was found to be significantly lower than the NIGHT one. HRI for PM_2.5_ doesn’t present significant diurnal variation. Considering the HB metric, no significant pattern could be derived for the whole region. The daily fluctuation of the population figures exhibits a higher influence on the resulting value. Hence, the daily number of commuters and the difference between DAY and NIGHT HBs rise with increasing degree of urbanization.

### Comparison with previous studies

Previous studies have assumed a static population for the health impact assessment [20–22]. Other studies have determined the personal exposure by considering the conditions in the occupied environments and the duration of the stay [32]. Our methodology adopted a dynamic population approach in order to better assess the exposure to air pollutants. The mobility dataset utilized captures the average travelling and commuting habits within a 5-days working week in Lombardy. This implies the loss of detail at individual level, that can be provided by wearables and mobile data [10, 14, 16]. On the other hand, this dataset allows an exposure evaluation from an indirect perspective. A similar approach was adopted by Reis et al. [43]. They found that the population-weighted exposure to yearly mean concentrations of NO_2_ and PM_2.5_, considering the workplace of the population, was 6.2% and 1% higher with respect to the exposure obtained considering the place of residence only. For O_3_, the exposure resulted to be 0.4% lower. The use of WSF 2019 provides an estimation of population presence by means of the FSA and allows the use of infiltration ratios applied to mask areas classified as “settled-building”. Furthermore, the WSF 2019, in combination with land use masks, provides a detailed population distribution in the geographical areas in the two scenarios that we considered. This opens a global perspective in health risk assessments.

Recent studies on the assessment of health risk from air pollution are predominantly limited to single cities, short time periods or single air pollutants [17, 26]. Dewulf et al. [10] presented a high spatial resolution and dynamic exposure assessment for Belgium but limited the study to NO_2_ and covered a time period of 48 h only. Ramacher et al. [25] introduced a dynamic population approach for exposure estimation for three cities in Northern Europe for the year 2012. This approach was subsequently adopted by Fenech and Aquilina [9] to study air pollution exposure in an area of Malta. However, it was limited to NO_2_ and made use of in-situ measurements from a single station to represent the variability of pollution over a large area. Our work provides a methodology to carry out a health risk assessment that is applicable to multiple air pollutants typically associated with health impairments. Furthermore, the geographical domain considered, includes several urban and rural environments in the whole region. In our study we addressed the health risk assessment of all-cause mortality associated with the long-term exposure to air pollutants. However, our approach can be easily replicated for the evaluation of further health impairments due to long-term exposure to air pollution, using suitable dose–response functions from medical literature. For example: mortality due to cardiovascular or respiratory diseases and hospitalization due to cardiovascular or respiratory diseases.

### Strengths and limitations

The CAMS European air quality reanalysis from an ensemble of nine chemical-transport models currently provides the most reliable and best systematically validated multiannual air pollution data set for the regional scale. The major drawback of CAMS Europe is its relatively coarse native spatial resolution. The interpolation method adopted to resample it on a finer grid was the bilinear one, as recommended by Stroh et al. [44]. In their study on pollution exposure modelling, they report that, for urban areas, the standard deviation between concentrations observed in a finer grid (100 × 100 m) and in a coarser one (1600 × 1600 m), resampled bilinearly to the same finer resolution, drops of about 78% when aggregating on a weekly basis with respect to the original hourly data, whilst it tends to zero for rural areas. The conclusion is that the accuracy of coarser datasets resampled bilinearly to a finer grid increases together with the aggregation time. In our study yearly aggregates were used. Moreover, De Ridder et al. [45] report that the yearly mean concentration of NO_2_ decreases by about 11% when the resolution is artificially reduced from 1 to 8 km and by about 21% when it is reduced to 15 km. Alternative datasets are in-situ measurements or Earth observation satellite data. However, in-situ data are already assimilated into the CAMS reanalysis and, although satellite-borne instruments enable the monitoring of NO_2_ [46, 47] and PM_2.5_ [48, 49], they are not yet capable of observing their diurnal cycles; due to the geometry of their Low Earth Orbit, they deliver just one or two overpasses per day. Hence, a combination of these observations with chemical transport models is still advisable.

The main challenge to the replicability of our method to other areas of interest is the acquisition of high-quality mobility data. However, initiatives are growing in this direction and new datasets of potential interest are being released and/or updated. These include the Mobility Data Specification, a data sharing platform that provides mobility data from multiple cities and transport agencies and the National Renewable Energy Laboratory, which provides mobility data on various cities, including information on traffic flow, speed and incidents, as well as data on public. In contrast, the WSF-2019 masks, utilized in this study for the first time for a health risk assessment, are already available worldwide and are currently being updated. The information on building use from OpenStreetMap is globally available. CAMS reanalyses with the resolution of 0.1 × 0.1 degrees are available for the European continent only. The CAMS global reanalyses dataset, with spatial resolution of 0.75 × 0.75 degrees, can be considered for a global application of the methodology. In this case, a higher uncertainty in the results is to be expected due to the loss of information in correspondence of urban hotspots. Future studies should address this aspect and provide a quantification of the introduced uncertainty and strategies for its compensation (Additional file 3: Table S3).

## Conclusions

With the COVID-19 pandemic and its early impact on the population of Lombardy, this region has become an area of interest for medical research. In this sense the COVID-19 pandemic substantiated the interaction between non-communicable and communicable diseases [50, 51]. Therefore, it is important to deliver a robust basis to assess the relationship between non-communicable diseases and air pollution. To our knowledge, this is the first study for Lombardy that combines dynamic mobility pattern and Earth observation data for a long-term exposure assessment from air pollution including O_3_, NO_2_ and PM_2.5_. Our approach demonstrates how the inclusion of mobility patterns and the differentiation between day and night population substantially impacts the exposure and health risk assessment. Our work aims at supporting public health protection and the United Nations Sustainable Development Goals by providing policy makers with an easy and replicable method to perform health risk assessment that accounts for population exposure and that is easily applicable to different geographical contexts. Our work can potentially support researches on causal relationships between air pollution and health outcomes exploiting an ecological design. We see the necessity of further studies to assess the performance of the application of our health risk assessment methodology to a global scale, possibly validating it with health data.

### Supplementary Information

**Additional file 1: Table S1.** Top-ten commuting areas disclosing the highest mean multiannual Health Risk Increase during the day and the night scenarios. When the same city is reported multiple times, a number is assigned next to the name, representing the districts of the major cities. As general criterion, the numbers are assigned with increasing order, starting from the city centre and moving radially and clockwise.

**Additional file 2: Table S2.** Top-ten commuting areas presenting the highest mean multiannual Health Burden during the day and in the night scenarios.

**Additional file 3: Table S3.** Difference in the number of inhabitants in Lombardy exposed different ranges of mean and maximum HRI of attributable to PM2.5, O3 and NO2. In brackets the values obtained using the upper and lower values of the 95% CI of the RR of mortality due to all causes.

## Data Availability

The datasets generated and/or analysed during the current study are available at the following links: settlement mask from the World Settlement footprint 2019 (https://geoservice.dlr.de/web/maps/eoc:wsf2019); land use data from OpenStreetMap (https://planet.osm.org/). Mobility data from Lombardy from Matrice Origine/Destinazione (https://www.dati.lombardia.it/Mobilit-e-trasporti/Matrice-OD2016-Passeggeri/tezw-ewgk); raster data with the commuting areas in Lombardy (https://www.dati.lombardia.it/Mobilit-e-trasporti/Shape-Matrice-OD2016-Passeggeri-Zone-interne/pium-6jqb). Datasets used and/or analysed during the current study on spatiotemporal aggregates of air pollutants concentration and the daily and night population of the commuting areas are available from the corresponding author on reasonable request.

## Abbreviations

CAMS: Copernicus atmosphere monitoring service
CI: Confidence interval
FSA: Fraction of settled area
GIS: Geographic information system
GPS: Global positioning system
HRI: Health risk increase
HB: Health Burden
IOR: Indoor/outdoor ratio
ISTAT: Italian National Institute of Statistics
MO/D 2014: Matrice origine destinazione
RR: Relative risk
WHO: World Health Organization
WSF: World settlement footprint
